# Determination of mutations in iron regulating genes of beta thalassemia major patients of Khyber Pakhtunkhwa, Pakistan

**DOI:** 10.1002/mgg3.1310

**Published:** 2020-06-25

**Authors:** Maryam Shah, Lubna Danish, Najeeb U. Khan, Fakhar Zaman, Muhammad Ismail, Mehfooz Hussain, Ruqiya Pervaiz, Aqib Iqbal

**Affiliations:** ^1^ Institute of Biotechnology and Genetic Engineering (Health Division) The University of Agricultural Peshawar Peshawar Pakistan; ^2^ Sulaiman Bin Abdullah Aba Al‐Khail Centre for Interdisciplinary Research in Basic Sciences (CIRBS) International Islamic University Islamabad (IIUI) Islamabad Pakistan; ^3^ Frontier Foundation Peshawar Pakistan; ^4^ Department of Zoology Islamia College Peshawar Peshawar Pakistan; ^5^ Department of Ophthalmology Leady Reading Hospital Peshawar Pakistan; ^6^ Department of Zoology AWKUM University Mardan Pakistan

**Keywords:** beta thalassemia major, G71D, H63D, *HAMP*, Hemochromatosis, Hepcidin, *HFE*

## Abstract

**Background:**

Hepcidin and hemochromatosis (*HFE*) are iron regulatory proteins that are encoded by *HAMP* and *HFE* genes. Mutation in either *HAMP* gene or *HFE* gene causes Hepcidin protein deficiency that can lead to iron overload in beta thalassemia patients. The aim of this research work was to study the presence of G71D mutation of *HAMP* gene and H63D mutation of *HFE* gene in beta thalassemia major and minor group to check the association of these mutations with serum ferritin level of beta thalassemia patients.

**Methods:**

The study was conducted on 42 beta thalassemia major and 20 beta thalassemia minor samples along with 20 control samples. The genotyping of both mutations has done by ARM‐PCR technique with specific set of primers.

**Results:**

Significant effect of G71D and H63D mutations was observed on serum ferritin level of thalassemia major group. The risk allele of *HAMP* G71D and *HFE* H63D was found with high frequency (48% and 49%, respectively) in beta thalassemia major than in control group. High genotypic frequency of *HAMP* and *HFE* gene mutation gene mutation was observed in beta thalassemia major than beta thalassemia minor and control group (7% and 9%, respectively).

**Conclusion:**

It can be concluded that both *HAMP* and *HFE* gene mutations show high frequency in beta thalassemia major patients and mean significant association between mutations and high serum ferritin level of beta thalassemia major patients but the nonsignificant results of Odd ratios showed that both mutations do not act as major risk factor in beta thalassemia major.

## Graphical abstract

We studied the presence of G71D mutation of *HAMP* gene and H63D mutation of *HFE* gene in beta thalassemia major and minor group to check the association of these mutations with serum ferritin level of beta thalassemia patients.

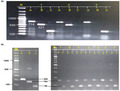

## 1 INTRODUCTION

Beta Thalassemia is an autosomal recessive disorder (Cao & Galanello, 2010), usually transmitted as recessive disorder from parents to offspring (Galanello & Origa, 2010) where there is less production or no production of Beta globin chains of hemoglobin (Hb; Weatherall & Clegg, 2001). There are four clinical types of Beta Thalassemia, two of them named as Carriers and Beta Thalassemia minor, which do not show any symptoms and are called silent types. The remaining two types are Beta Thalassemia major and intermedia, which require medical attention that needs blood transfusion for survival (Cao & Galanello, 2010). The severe types of Beta Thalassemia are usually cause by mutant Beta globin gene either in homozygous condition or heterozygous condition (Wafaa, 2017). Usually the diagnosis of Beta Thalassemia major occurs at age 1–24 months. The growth of patient get slow and the skin of the patient became pale and can suffer from fever, diarrhea, abdominal enlargement due to splenomegaly and enlarge liver (Galanello & Origa, 2010).

Being common inherited disorder in Pakistan an estimate there is 50,000–100,000 patients suffering from beta Thalassemia major and about 6,000 new born each year (Ali et al., 2015; Baig et al., 2006). Beta Thalassemia major patients face various complications that includes anemia, ineffective erythropoiesis, and iron overload (Gardenghi et al., 2010) that occurs due to regular blood transfusion as well as the intake of iron containing diet. The iron deposition in different tissues and organs (Patel et al., 2012) can leads to many other complications like hearts problems, dilated cardiomyopathy, arrhythmias, liver problems (cirrhosis and fibrosis), hyper pituitarism, diabetes mellitus, and decrease production of parathyroid, thyroid, pituitary, and adrenal gland hormones (Marengo‐Rowe 2007) .

Study showed that in case of Beta Thalassemia, there is a protein named Hepcidin has primary role in iron metabolism (Nemeth & Ganz, 2009). Hepcidin is a protein consists of 25 amino acids that are encoded by gene named *HAMP* (Nemeth et al., 2004). Hepcidin regulates absorption of iron from small intestine and recycle with the help of macrophages (Nemeth et al., 2003; Pak, Lopez, Gabayan, Ganz, & Rivera, 2006). Hepcidin functions by degrading Ferro‐protein (Nemeth et al., 2004) that export iron by expressing on duodenum erythrocytes, liver cells, and macrophages (Gardenghi et al., 2010). Hepcidin expression is upregulated in case of iron overload (Pigeon et al., 2001) and inflammation (Nemeth et al., 2003; Nicolas et al., 2002; Wrighting & Andrews, 2006). The low expression level of Hepcidin is usually seen whether there is mutation in *HAMP* gene or a gene named Hemochromatosis (*HFE*), that codes for *HFE* protein in case of *HFE* disease (Le Gac & Férec, 2005). Moreover, deficiency of Hepcidin also leads to iron overload in case of Beta Thalassemia major (Nemeth, 2010). The G71D mutation of *HAMP* gene presents in between 4 and 8 cysteine of Hepcidin structure, due to which the neutral Glycine is converted into acidic Aspartic acid. This mutation is supposed to be one of the factors of iron overload (Jacolot et al., 2004). *HFE* protein is transmembrane protein, which help to decrease the affinity of Transferrin receptor to transferrin loaded with iron, by binding to transferrin receptor (Feder et al., 1998). In case of H63D mutation in *HFE* gene, not only absorption of iron increases, but also decreases the affinity between transferrin and its receptor and acts as a major factor of iron overload. In case of H63D mutation, high concentration of iron absorption takes place from small intestine. As Hepcidin regulates the absorption of iron from small intestine, so *HFE* gene mutation affects the function of Hepcidin. *HFE* gene mutation also decrease the affinity between Hepcidin and FP1 (an iron export protein), resulting high iron deposition in tissues (Dasgupta, Roy, & Sinharay, 2017). It is hypothesized that there could be relation between G71D of *HAMP* and H63D of *HFE* gene mutations with iron overload in case of Beta Thalassemia major in KP‐population. Therefore, the purpose of our study is to determine the presence of *HAMP* and *HFE* gene mutations in beta Thalassemia major patients in KP population and its relation with iron overload.

## 2 MATERIALS AND METHODS

### 2.1 Blood sampling

The study was conducted on 42 beta thalassemia major patients registered at Frontier Foundation Hospital, Peshawar and regularly attending the hospital for blood transfusion. The HB level of all the beta Thalassemia major patients was in the range of 3–5 µl/dl, while the age range was 1–23 years with mean age of 9.19 years. Total number of cases consists of 21 male (50%) and 21 female patients (50%). The control samples included 20 Beta Thalassemia minor and 20 healthy individuals. The age range of Beta Thalassemia minor subjects was 19–35 with mean age of 25.9 years, while age range of healthy controls subjects was 2–19 years with mean age of 9.6 years. Informed consent has been approved from all the subjects and their family. From all the subjects 4 ml blood samples were taken in EDTA tubes for molecular study keeping in notice the standard biosafety protocol.

### 2.2 Measurement of serum ferritin level

The Serum Ferritin level of 42 beta thalassemia major patients was measured by sandwich ELISA (Enzyme Linked Immunosorbent Assay) technique (Kazmi, Mansoor, Almani, & Zafar, 2017).

### 2.3 DNA isolation

All the samples (Patients and Controls) were processed for genomic DNA extraction using nonenzymatic/salting out method (Suguna, Nandal, Kamble, Bharatha, & Kunkulol, 2014).

### 2.4 PCR amplification

For genotyping T‐ARMs PCR was carried out, briefly, four primers of each SNP were designed, Forward Outer, Forward Inner, Reverse Outer, and Reverse Inner. Forward and reverse primers of G71D (*HAMP*) were 5ʹ‐ATGCAGGGAGGTGTGTTAGGAGG‐3ʹ (Fo), 5ʹ‐CCATCTGCATTTTCTGCTGCGG‐3ʹ (Fi), 5ʹ‐TGCAAGGCAGGGTCAGGACAAGC‐3ʹ (Ro) and 5ʹ‐CACTTTGATCGATGACAGCAGT‐3ʹ (Ri). While primers for H63D (*HFE*) were as follow; 5ʹ‐ACATGGTTAAGGCCTGTTGC‐3ʹ (Fo), 5ʹ‐CCAGCTGTTCGTGTTCTATGATC‐3ʹ (Fi), 5ʹ‐GCCACATCTGGCTTGAAATT‐3ʹ (Ro) and 5ʹ‐GGCTCCACACGGCGACTCTCATC‐3ʹ (Ri). Outer and Reverse Inner primers were used to amplify gene segment containing the SNP, Forward outer and Reverse Inner were used to amplify mutated nucleotide and Forward Inner and Reverse Outer were used to amplify wild nucleotide. PCR mixture of 25 µl was made consisting of 12 µl master mix, 1 µl of each forward and reverse primer, 3 µl of template DNA, and 7.5 µl of ddH_2_O. The PCR amplification conditions were; initial denaturation 95ºC for 5 min, proceeding with 35 cycle of denaturation at 95ºC for 30 s, annealing at 59ºC for 30 s, extension 72ºC for 30 s, and final extension at 72ºC for 5 min. The amplified PCR products of both genes were run on 1% of agarose gel.

### 2.5 Statistical analysis

Statistical analysis was carried out using SPSS software version 2.0. Descriptive statistical method was used for demographic data analysis with *p* < .05 was considered significant. The comparison of genotypes from two groups (beta thalassemia major group and control beta thalassemia minor and control group, beta thalassemia major and beta thalassemia minor) were done by obtaining Odd ratios and 95% CI value using online software named Medcalc Odd ratio Calculator. The *p* < .05 were considered nonsignificant.

## 3 RESULTS

**Figure 1:**
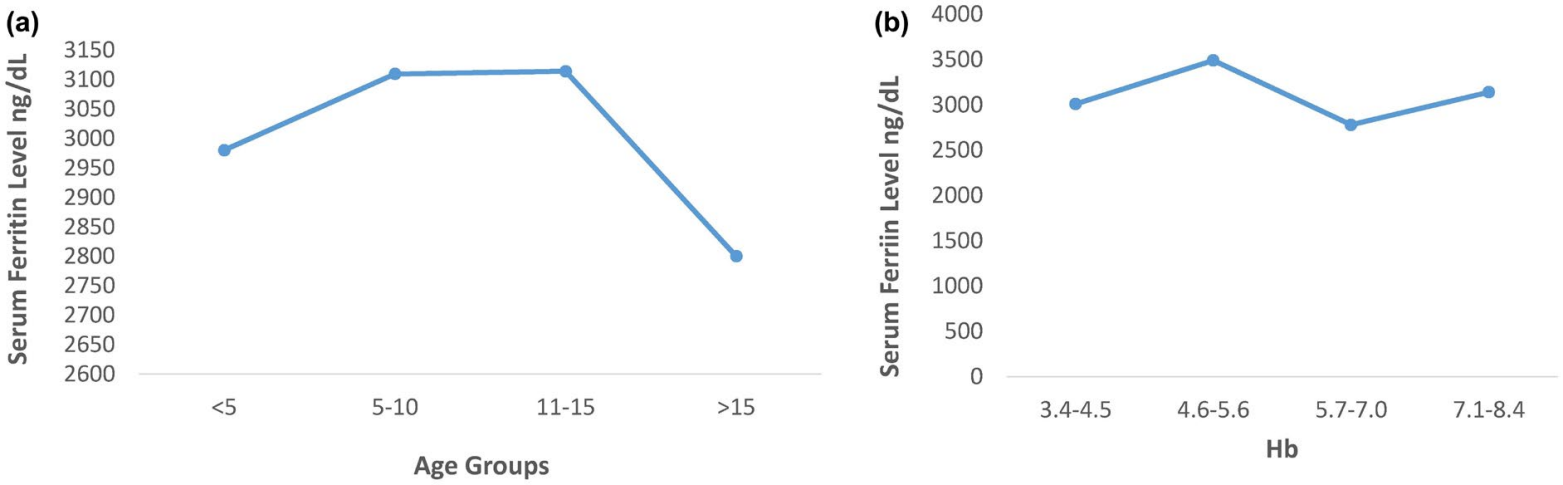
Effect of age and hemoglobin level on Serum Ferritin level

Serum Ferritin level was calculated by ELISA technique. The serum ferritin level of all the cases was in range of 2000–4000 g/dl with mean value of 3,080.9 g/dl. The highest number of patients (26%) had Serum ferritin level between 3500 and 4000 g/dl. While 21% had 2500–2900 g/dl, 19% had 3000–3400 g/dl, and 5% had between 2000 and 2400 g/dl. The study was conducted on 42 blood samples of Beta Thalassemia major patients with age 1–23 with mean age 9±0.654. Age was nonsignificantly associated with the iron level of Beta Thalassemia patients (*P*0.857) while significant association (*P *= .05) was found between HB and serum ferritin level. Dropdown can be seen in serum ferritin level with increase in Hb level (Figure 1).

The statistical analysis indicated no significant effect of gender on serum ferritin level (*p* = .47), but females’ patients showed low serum ferritin level (3,019.05 ± 104.794) than male (3,142.86 ± 133.376; Table 1). The coinfections were also found among these patients. Out of 42 patients, 10 patients were coinfected with Hep C (24%) and 32 have not any infection (76%). Although infection did not show significant effect on serum ferritin level (*p* = .901), no significant difference has obtained between serum ferritin level of infected and noninfected patients, but patients with Hep C infection showed high serum ferritin (3,100 ± 167.332) than patients without infection (Table 1). On the contrary, male patients showed low serum ferritin level (2,900.00 ± 285.774) when they are infected while in case of females; low serum ferritin level was observed if they were infected (2,933.33 ± 118.590; Table 2).

**Table 1:** Effect of hepatitis C (Hep C) infection on serum ferritin level

	Mean serum ferritin	*SEM*
Gender		
Male	3,142.86	133.376
Female	3,019.05	104.794
Hep C infection		
+	3,100	167.332
−	3,075	98.987

**Table 2:** Correlation between gender and Infection with serum ferritin level

Gender	Mean	*N*	*SD*	*SEM*
Male				
0	3,200.00	17	622.495	150.977
1	2,900.00	4	571.548	285.774
Total	3,142.86	21	611.205	133.376
Female				
0	2,933.33	15	459.296	118.590
1	3,233.33	6	504.645	206.020
Total	3,019.05	21	480.228	104.794

All the patients were on regular blood transfusion; seven patients (17%) received blood after a week, 6 (14%) after 10 days, 13 (31%) after 2 weeks, 7 (17%) after 3 weeks, 8 (19%) receive after 4 weeks, and 1 (2%) patient was receiving blood transfusion after each 5 weeks. The data indicated that most of the patients were receiving blood transfusion after 2 weeks.

Beta thalassemia major patients suffer from various kinds of complications. It was found that mostly patients suffer from body pain (48%) that can increase with the age. The body pain has mostly seen in patient's age range of 7–11 years. The second complication mostly observed in beta thalassemia major patients was temperature. Highest number of patients (40%) complaining about temperature was of age range 7–11 years. The third highest occurring complication was joint pain observed in 14% of patients in which mostly patients were within age of 7–11 years. Some of the patients have also undergone Splenomegaly (10%), pancreatitis (7%), and hepatomegaly (2%) mostly belongs to age group 7–11. Splenectomy was also observed in 7% patients of age >11. Some patients (5%) belongs to age group <7 and <11 had complained about Cardiac problem, while there were also complications like headache (7%), chest problems and nose bleeding (5%) in age group 7–11 and <7. Stomachache and Blood stool problem has also observed in 2% of patients (Table 3).

**Table 3:** Clinical complications of beta thalassemia patients

Complication	Total patients	Patients *n* with complications, %	Age group		
			<7 *n* (%)	7–11 *n* (%)	>11 *n* (%)
Body pain	42	20 (48)	3 (15)	10 (50)	7 (35)
Splenomegaly	42	4 (10)	2 (50)	2 (50)	0 (0)
Splenectomy	42	3 (7)	1 (33)	0 (0)	2 (67)
Pancreatitis	42	3 (7)	0 (0)	2 (67)	1 (33)
Cardiac problem	42	2 (5)	1 (50)	0 (0)	1 (50)
Hepatomegaly	42	1 (2)	0 (0)	1 (100)	0 (0)
Temperature	42	17 (40)	4 (23)	9 (53)	4 (23)
Joint pain	42	6 (14)	1 (17)	3 (50)	2 (33)
Blood stool	42	1 (2)	1 (100)	0	0 (0)
Chest problems	42	2 (5)	2 (100)	0 (0)	0 (0)
Headache	42	3 (7)	1 (33)	2 (67)	0 (100)
Nose bleeding	42	3 (7)	0 (0)	2 (67)	1 (33)
Stomach ache	42	1 (2)	0 (0)	1 (100)	0 (0)
Total	42		16	32	18

**Figure 2:**
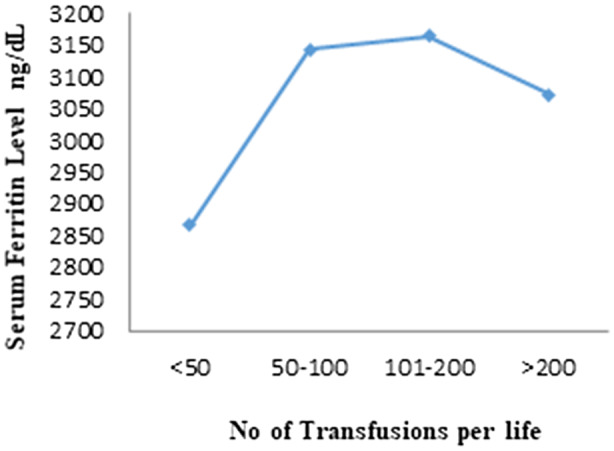
Effect of total blood transfusions on serum ferritin level

Most of the patients (45%) had received total blood transfusion between 100 and 200 times, while 21% patients had received less than 50 transfusions in their whole lifespan. The patients received blood transfusions between 50 and 100 and more than 200 were 17%. Nonsignificant relation was obtained between serum ferritin level and total number of transfusions (*p* = .608). Figure 2 showed that level serum ferritin level increased with increase in blood transfusions number while a decrease in serum ferritin level was observed during number of blood transfusion >200.

All the patients were on iron chelation therapy in which, 10/42 (24%) was on Tab. asunra 400 mg (1), 13/42 (31%) was taking Tab. Desirox 400 mg, 1/42 (2%) on Tab. Asefed 500 mg, 12/42 (28%) on inj. Desferal 500 mg, 5/42 (11.9%) on Cap. Kelfer 500 mg, and 1/42 (2%) was taking Tab. Oderox 500 mg. No significant effect of Iron chelation medicines was observed on serum ferritin (*p* = .552).

### 3.1 Genotype distribution of G71D (*HAMP)* and H63D (*HFE*)

**Figure 3:**
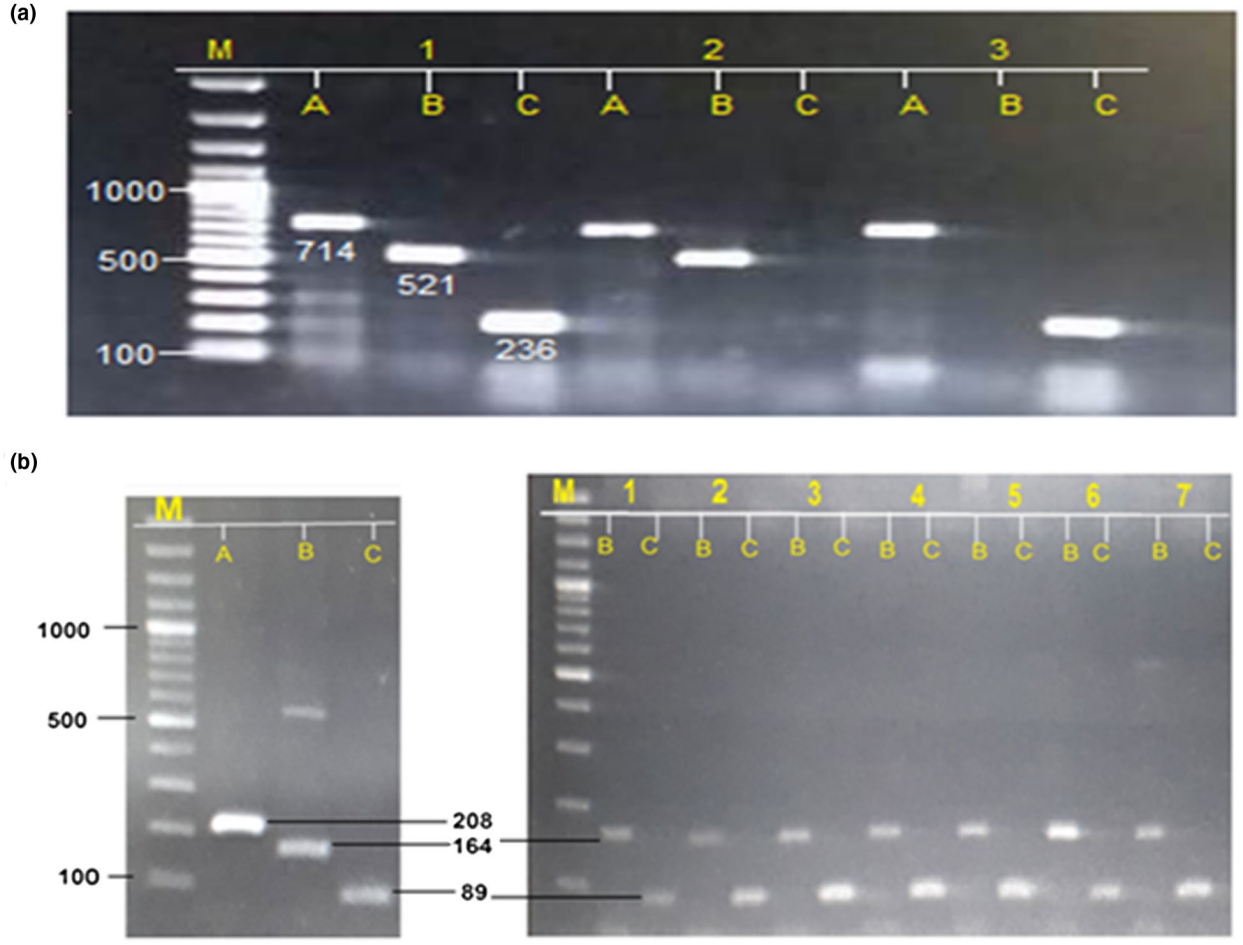
Samples gel picture for *HAMP* (G71D) and *HFE* (H63D)

ARM‐PCR technique was performed for all blood samples (beta thalassemia major group, beta thalassemia minor and control group). In this study, significant association has obtained between serum ferritin level of beta thalassemia major patients and genotypes of both genes (*p*=.00, .05, respectively). In case of *HAMP* gene, highest serum ferritin level was observed in heterozygous genotype GA of than homozygous mutant genotype AA, on the contrary, the homozygous mutant genotype GG of *HFE* gene showed high level of serum ferritin than heterozygous genotype CG (Figure 3; Table 4).

**Table 4:** Association of genotypes and serum ferritin level according to Mean ± *SD*

Genotype	Mean ± *SD*	*p* value
G71D (*HAMP*)		
GG	2,765.96 ± 74.332	.000
GA	3,163.99 ± 29.071	
AA	2,839.29 ± 115.165	
H63D (*HFE*)		
CC	3,138.95 ± 39.348	.005
CG	3,047.57 ± 36.528	
GG	3,400.00 ± 27.434	

In case of beta thalassemia major case, 5 (12%) showed homozygous wild genotype, 34 (81%) showed heterozygous, 3 (7%) showed homozygous mutant genotype of G71D mutation, on the contrary, 5 (12%) showed homozygous wild genotype, 33 (78%) showed heterozygous, and 4 (9%) showed homozygous mutant genotype for H63D mutation. No patient was observed having both mutations in homozygous state. The allelic percentage G, A, C, and G allele were 52%, 48%, 51%, and 49%, respectively. In case of beta thalassemia minor group, 2 (10%) showed homozygous wild genotype, 17 (85%) showed heterozygous, 1 (5%) showed homozygous genotype of G71D mutation. Moreover, 3 (15%) homozygous wild genotype, 16 (80%) showed heterozygous, and 1 (5%) homozygous mutant genotypes for H63D mutation. The frequency of G, A, C, and G allele were 52%, 48%, 55%, and 45%.

The healthy control samples carried 3 (15%) homozygous wild genotype, 16 (80%) heterozygous, and 1 (5%) homozygous mutant genotype of G71D mutation, while in case of H63D mutation, there were 2 (10%) homozygous wild genotype, 17 (85%) heterozygous, and 1 (5%) homozygous mutant genotype. The frequency of G, A, C, and G allele were 55%, 45%, 53%, and 47%.

The calculated odd ratios and frequencies of both SNPs in beta thalassemia major, minor, and control group are given in Table 5. In case of G71D (*HAMP*), same frequency of the risk allele A was observed in both beta thalassemia major patients and beta thalassemia minor. The mutation did not show significant association with beta thalassemia major group (*p* = .787) and beta thalassemia minor group (*p* = .8226).

**Table 5:** Allele and genotype distribution of SNPs (G71D & H63D) in beta thalassemia major, beta thalassemia minor and control group

Allele/genotype	Patients, *n* (%)	Control, *n* (%)	Odd ratios	95% CI	*p* value
Allele and genotype distribution of HAMP (G71D) in patients and control group					
G	44 (52)	22 (55)	Reference		
A	40 (48)	18 (45)	1.1111	0.5218–2.3661	.7847
GG	5 (12)	3 (15)	Reference		
GA	34 (81)	16 (80)	1.2750	0.2707–6.0060	.7587
AA	3 (7)	1 (5)	1.8000	0.1237–26.1973	.6670
Allele and genotype distribution of HFE (H63D) in patients and control group					
C	43 (51)	21 (53)	Reference		
G	41 (49)	19 (47)	1.0539	0.4959–2.2394	.8915
CC	5 (12)	2 (10)	Reference		
CG	33 (78)	17 (85)	0.7765	0.1361–4.4288	.7758
GG	4 (9)	1 (5)	1.6000	0.1036–24.7047	.7364
Allele and genotype distribution of HMP (G71D) in beta thalassemia minor and control group					
G	21 (52)	22 (55)	Reference		
A	19 (48)	18 (45)	1.1058	0.4590–2.6641	.8226
GG	2 (10)	3 (15)	Reference		
GA	17 (85)	16 (80)	1.5938	0.2348–10.8172	.6333
AA	1 (5)	1 (5)	1.5000	0.0554–40.6353	.8096
Allele and genotype distribution of HFE (H63D) in beta thalassemia minor and control group					
C	22 (55)	21 (53)	Reference		
G	18 (45)	19 (47)	0.9043	0.3754–2.1787	.8226
CC	3 (15)	2 (10)	Reference		
CG	16 (80)	17 (85)	0.6275	0.0924–4.2587	.6333
GG	1 (5)	1 (5)	0.6667	0.0246–18.0601	.8096

*HFE* genotype GG was observed in high frequency in patients than minor and control group. The table showed that risk allele G did not show significant association with beta thalassemia major group (*p* = .8915) and beta thalassemia minor group (*p* = .8226).

The genotypic comparison of both SNPs between beta thalassemia major and beta thalassemia minor group has given in Table 6. In case of G71D (*HAMP*), high frequency of homozygous mutant genotype was obtained in beta thalassemia major group than beta thalassemia minor group. In case of risk allele A, same frequency was observed in both groups. The result of statistical analysis showed that no significant difference of mutant allele A between beta thalassemia major and beta thalassemia minor group (*p* = .9901).

**Table 6:** Allele and Genotypic comparison between beta thalassemia major and minor group

Allele/genotype	Major, *n* (%)	Minor, *n* (%)	Odd ratios	95% CI	*p* value
Comparison of allele and genotypic distribution of HMP (G71D) between major & minor group					
G	44 (52)	21 (52)	Reference		
A	40 (48)	19 (48)	1.0048	0.4727–2.1356	.9901
GG	5 (12)	2 (10)	Reference		
GA	34 (81)	17 (85)	0.8000	0.1404–4.5585	.8016
AA	3 (7)	1 (5)	1.2000	0.0733–19.6324	.8983
Comparison of allele and genotypic distribution of HFE (H63D) between major & minor group					
C	43 (51)	22 (55)	Reference		
G	41 (49)	18 (45)	1.1654	0.5474–2.4812	.6914
CC	5 (12)	3 (15)	Reference		
CG	33 (78)	16 (80)	1.2375	0.2624–5.8358	.7877
GG	4 (9)	1 (5)	2.4000	0.1752–32.8806	.5121

Homozygous wild genotype of H63D mutation CC was observed in high frequency in beta thalassemia minor group than beta thalassemia major group, while homozygous mutant genotype GG showed high frequency in beta thalassemia major group (9%). The risk allele G was obtained with high frequency in beta thalassemia major group. No significant difference was observed between beta thalassemia major and beta thalassemia minor group in case of mutant allele G (*p* = .6914).

## 4 DISCUSSION

It is necessary for a beta thalassemia major patient to get blood transfusion regularly that leads to iron overload which body cannot excrete (Cappellini et al., 2007). About 250 mg iron is present in a single unit of red blood cells bag, which is to be transfused (Ozment and Turi, 2009). On the contrary, body can only excrete 1 mg of iron in a day. So if a patient is receiving 25 units of red blood cells bag a year, there will be accumulation of 5 grams of iron per year. Included increase absorption of iron take place by intestinal in beta thalassemia major patients (Mishra and Tiwari, 2013). Instead of regular use of iron regulation medicines, there patients suffer from iron overload. Iron starts depositing in parenchyma tissues cells of body during the first year of transfusion in beta thalassemia major patients (Taksande, Prabhu, & Venkatesh, 2012). The level of serum ferritin changes with age. At the time of birth, the concentration is usually low, while increase occurs at early 2 months of life, and decrease starts throughout infancy later (Domellof, Dewey, Lonnerdal, Cohen, & Hernell, 2002). At the start of adulthood, there is high concentration of serum ferritin in males than females (Cappellini et al., 2007). In these patients, iron level should regular be monitor in order to find out the toxicity caused by excess of iron and side effects of excessive use of iron chelation medicines (Porter and Davis, 2002). Otherwise, excess iron in the body can harm the body cells and leads to serious damage to organs and tissues like heart problems, diabetes, hypogonadism, cirrhosis (Melchiori, Gardenghi, & Rivella, 2010), and body pain which occurs due to low Hb level, iron overload, or low mass of bones. Study indicated no significant association of iron chelation medicines or low Hb level with pain, but low bone mass has considered to be the reason of pain in Thalassemia patients. With increase in age, the pain becomes severe in different body parts (Haines et al., 2013). In this study, most of the patients were suffering from body pain, joint pain while rest of complications was not that much common. That most of complications were found in older age that indicates that pain and complications increase with age (Table 3). The standard level of serum ferritin considered for beta thalassemia major patients is 1000 mg/L (Shah, Trehan, Das, & Marwaha, 2014). After serum ferritin reaches the level 1000 mg/L, usually after receiving 11–12 blood transfusions, the patients have to starts iron chelation therapy (Mishra and Tiwari, 2012). In the current study, iron chelation medicines and total number of blood transfusion did not show significant impact on serum ferritin level (*p* = .5520, 0.608) Patients that are suffering from hepatitis C (Hep C) infections can also cause iron overload by deposition of iron in liver cells and reticuloendothelial cells (Hörl and Schmidt, 2014; Price and Kowdley, 2009). Studies showed that Hep C virus decrease the Hepcidin protein expression by activating transcription of SMAD signaling pathways and signal transducers (Zou and Sun, 2017). In the current study, besides nonsignificant relation with serum ferritin, high serum ferritin level was found in patients suffering from Hep C infection. Moreover, in case of infection, male patients showed low serum ferritin level while female patients showed high serum ferritin level with infection (Table 1 and 2).

Studies suggested that G71D mutation of *HAMP* gene act as modifier in iron overload diseases (Altès et al., 2009; Biasiotto et al., 2004; Jacolot et al., 2004; Merryweather‐Clarke et al., 2003). Although how this mutation affects the function of Hepcidin protein is unknown (Jacolot et al., 2004). On the contrary, H63D mutation can also affect the serum ferritin level in beta thalassemia major patients (Melis et al., 2002). In this study, G71D and H63D mutation showed significant impact on serum ferritin level (Table 4), high average mean of serum ferritin has obtained with homozygous mutant condition than homozygous wild genotype in case of G71D mutation. The odd ratios indicated strong association of homozygous mutant genotype AA with Beta Thalassemia major group while in case of also Beta Thalassemia minor group, strong association of heterozygous genotype GA was obtained. High mean of serum ferritin was observed with homozygous mutant genotype than homozygous wild genotyping case of H63D mutation. While as a risk factor, H63D mutation did not show significant relation with beta thalassemia major patient or beta thalassemia minor group. The nonsignificant effect can be link with the high heterozygosity. As study showed that H63D mutation in homozygous condition has relation with iron overload, where change in normal *HFE* pathway occur due to H63D mutation in mice, that leads to iron overload (De Diego, Opazo, Murga, & Martínez‐Castro, 2007). Moreover, in beta thalassemia major or minor cases, this mutation in heterozygous condition does not impact the serum ferritin level (Piperno et al., 2000; Yamsri et al., 2007).

## 5 CONCLUSION

The G71D and H63D mutation of *HAMP* and *HFE* gene are frequently found in beta thalassemia major patients. The genotype was also observed in beta thalassemia minor group. Although significant effect of both mutations was obtained on serum ferritin level but they did not act as major risk factor in beta thalassemia major patients. But the frequent presence of G71D and H63D mutation in beta thalassemia major patients indicated the possible association between SNPs and iron regulation pathway. This increases the value to screen *HAMP* and *HFE* mutations in beta thalassemia major patients in order to change iron overload treatment.

## CONFLICT OF INTEREST

None declared.

## AUTHORS CONTRIBUTION

MS and MH collected the samples and performed the experimental work. NK, RP, and AI designed the study and methodology. LD wrote the manuscript and NK proofread the final version. MI conducted all the statistical analysis. All authors reviewed the final manuscript.

## Data Availability

The data used to support the findings of this study are included within the article; however, the raw data files are available from the corresponding author upon request.
